# Supplementary material for: Identifying primary-care features associated with complex mental health difficulties
Source: PLoS One. 2025 May 8;20(5):e0322771. doi: 10.1371/journal.pone.0322771 (PMC12061163; doi:10.1371/journal.pone.0322771)
Supplement: Attachment — Submitted filename: Response to Reviewers.docx [file pone.0322771.s006.docx]

|  |  | School of Medicine and Population Health  The University of Sheffield  Barber House  387 Glossop Road  Sheffield S10 2HQ |
| --- | --- | --- |
| 8^th^ Apr 2024 |  | **Telephone:** +44 (0) 79 030 636 92  **Email:** ciaran.mcinerney@sheffield.ac.uk |

Dear, Reviewers.

We thank you for your time and expertise that you committed to reviewing our submission. We have amended our manuscript in response to your insightful comments. We provide a response to each of your comments, below.

Yours sincerely,

Ciarán McInerney

in co-authorship with the UnSeen project team

Reviewer #1: This is an interesting attempt to identify cases of complex mental illness in medical records. While not completely successful in doing so, it does add to the literature in this area. However, I have concerns regarding the inclusion and exclusion criteria, in particular the way that certain items were operationalised. I feel "chronic depression" will not have captured many with chronic depression; while "severe mental illness" excluded many incentivised terms for psychotic illness, and only four antipsychotics were considered. I feel these issues require some more detailed justification and explanation in the text. Specific comments in relation to these points and others are below. There were also quite a few editorial issues, such as the lack of graph titles/footnotes, mislabelled supplements and tables, and the broken link to github.

1. Line 63: The prevalence appears to vary substantially in the WHO study with >7% in the US And Colombia and only 2% in Western Europe. This study in the UK suggests around 4% which may be more accurate: <https://pubmed.ncbi.nlm.nih.gov/16648528/>

We thank the reviewer for directing us to the additional reference. We have included it in the manuscript.

2. Lines 68-72: This study uses THIN, which does not have full UK coverage. The methods of this paper state at the time of the study it had records for 744 GP practices, accounting for approximately 6% of the UK population. Therefore, if you assume a population of 60 million, the denominator should be 6% of this, and this study identified around 1.3% of patients with codes for PD (46,210 / 3,600,000), rather than 0.008%. Likewise, the Norwegian study was only looking at diagnoses made in a one year period, and so was not looking at the prevalence of PD in the sample, but those who had a consultation for PD in that one year.

We thank the reviewer for spotting the particular sampling bias of T.H.I.N. that is noted on page 2 of the cited UK study. For clarity, we arrived at our rounded value of 0.08% by using the 46,210 count of those recorded with personality disorder as the numerator, and 60 million as the denominator. We agree that the reviewer’s suggested denominator is more appropriate, so we have changed the estimated prevalence.

Regarding the Norwegian study, we have amended the text to clarify that the prevalence is a one-year incidence rather than a prevalence.

3. Line 73: This study is based in the US which is a very different HER system to ours. Depression (as a clinical code or prescription of antidepressants) is recorded relatively well in the UK, it is the determining whether it is a persistent depression that is harder. This may be a better reference for how much is recorded for depression/PTSD in the UK, though it doesn’t detail persistent depression: <https://bmcmedinformdecismak.biomedcentral.com/articles/10.1186/s12911-023-02296-z/tables/3>

We thank the reviewer for directing us to the alternative reference.

4. Line 81: Another really important reason the authors may want to consider is that as of Jan ’24 NHS England advise “ICSs are strongly encouraged to consider the physical health needs of all people severely affected by their mental illness…This includes, but is not limited to, those with a diagnosis of personality disorder, eating disorder or severe depression, and people with mental health rehabilitation needs”. However, without these diagnoses recorded in primary care the GP will be unable to act upon this: <https://www.england.nhs.uk/long-read/improving-the-physical-health-of-people-living-with-severe-mental-illness/#the-smi-register-and-smi-annual-physical-health-checks>

We thank the reviewer for highlighting the advice given in January 2024. Our draft missed this information because the study was completed in 2023. We now refer to this important publication in our Discussion, to set the context.

5. Line 110: Note, this link does not currently work so I have been unable to review anything on the github.

We apologise for the inconvenience. We have contacted Connected Bradford, who have now made all study files public.

6. Line 161: This data is in S2 Table, not S1.

Apologies for this error, and thanks to the reviewer for spotting it. We have amended it.

7. Table 1: This data is in S2 Table, not S3

Apologies for this error, and thanks to the reviewer for spotting it. We have amended it.

8. Table 1: dementia is listed as an exclusion but not justified in the text.

Thanks for pointing out our oversight (this had been included in earlier documents). We have now added our justification to the others.

9. Line 161 you state “a SNOMED-CT diagnostic code for caseness (see link associated with "Mental disorder | SCTID: 74732009 + child codes" but then in Table 1 you have much more stringent inclusion criteria. Referring to Figure 1 I see that this is a stepped approach, and assume a case is a patient with a diagnosis as per the inclusion in Table 1? This could be clearer in the text

Apologies for this error, and thanks to the reviewer for spotting it. We have made the following amendments: the explanation of the definition of our study population now includes the note about “Mental disorder | SCTID: 74732009 + child codes” in S2 Table; the explanation of our caseness now reads “…defined by having 1) at least one SNOMED-CT diagnostic code from our inclusion list, excluding those from our exclusion list…(Table 1)”.

10. Controls: Were controls those that met neither the diagnosis or the medication criteria, or could they meet one criteria but not the other? Were controls able to have diagnoses such psychotic disorders? We know a lot of patients with codes for psychosis go on to receive a code of schizophrenia, and so I wonder whether removing them may have given a “cleaner” control group. GPs in England are incentivised to keep a record of patients with SMI (including schizophrenia and bipolar disorder but also other psychoses – e.g. schizoaffective schizophrenia, first episode psychosis). Because of this, people with any of the SNOMED codes included in the NHS incentivised to keep a record of patients with SMI (including schizophrenia and bipolar disorder but also other psychoses – e.g. schizoaffective schizophrenia, first episode psychosis). Because of this, people with any of the SNOMED codes included in the NHS incentivised definition are likely to be well recorded, but not all meet your criteria of SMI. The Hardoon paper from your introduction found that of people with personality disorder, 20% had an SMI (schizophrenia, bipolar disorder, other psychosis) diagnosis, so the cross-over between SMI and PD needs some careful thought. Excluding them from cases removes potentially quite a few people with PD, while not fully excluding them from controls (i.e. including those with other psychoses) may be contaminating your control group

We thank the reviewer for this comment, which we think is related to comment 22. We decided to replace our codelists for bipolar and schizophrenia with a single codelist for the NHS ResSet for MH-COD, which specifies psychosis, schizophrenia and bipolar affective disease codes (<https://www.opencodelists.org/codelist/nhsd-primary-care-domain-refsets/mh_cod/20210127/#tree>). The ramifications were of this change are:

- a redacted-and-rounded count of 5,500 patient records contained the MH-COD codes, whereas only 4,040 records contained either bipolar (1,860) or schizophrenia (2,180).
- The percentage of caseness among those with mental ill-health in the Connected Bradford dataset dropped to 1.96% from 2.16%.
- The percentage of patient records with at least one diagnosis dropped to 2.6% from 2.9%.
- The percentage of patient records with at least one medication dropped to 61.5% from 62.6%.
- The caseness variable’s entropy dropped to 0.099 nats from 0.106 nats.
- The caseness variable's entropy dropped to 14.3% of its theoretical maximum, from 15.4% of its theoretical maximum.
- The accuracy (a.k.a. hit rate) of a rule classifying all study-population records as meeting our definition of complex mental health difficulties dropped from 2.218% to 2.029%.
- The accuracy (a.k.a. hit rate) of a rule classifying none of study-population records as meeting our definition of complex mental health difficulties increased from 97.782% to 97.971%.
- The odds that record did not meet our definition of complex mental health difficulties increased from 44 to 48-times as likely.

We also recognise the cross-over between SMI and PD, both in terms of shared risk factors and overlapping symptoms and impairment. Nevertheless, we exclude selective SMI codes because one of the potential predictors we are using is antipsychotic medication in the absence of psychosis. Pragmatically also, people with co-morbid PD and psychosis are likely to be visible to specialist mental health services rather than “unseen”.

11. Table 1: Could the authors justify their selection of antipsychotics? I was interested that only four were chosen. While quetiapine, olanzapine and risperidone are very commonly prescribed, the paper by Hardoon et al lists chlorpromazine, aripiprazole and haloperidol as more commonly prescribed to patients with personality disorder than flupentixol, while more recent analysis of patients with SMI shows that aripiprazole is now the second most commonly prescribed.

We thank the reviewer for pointing out this omission. We have included the following medications in the updated scripts: chlorpromazine, aripiprazole and haloperidol. Note: we first applied the suggested change to the exclusion codelist, and have presented the ramifications previously in our response. The ramifications of adding these medications to the caseness definition are:

- a redacted-and-rounded count of patient records contained medications for psychosis and related medications increased from 7,610 to 8,430. This did not change the percentage of records meeting this component of our caseness definition, at two decimal places.
- The percentage of caseness among those with mental ill-health in the Connected Bradford dataset remained at 1.96%. This was unsurprising given that we have noted how the diagnoses criterion has been the limiting component of our definition.
- The count of records meeting our criteria for caseness remained unchanged at 3,040.
- The percentage of patient records with at least one medication remained at 61.5%, but the redacted-and-rounded count decreased from 68,210 to 68,060. This indicates that the addition of the medications to the list has updated information on polypharmacy, even though it has not identified any new records.
- The caseness variable’s entropy remained at 0.099 nats.
- The caseness variable's entropy remained at 14.3% of its theoretical maximum.
- The accuracy (a.k.a. hit rate) of a rule classifying all study-population records as meeting our definition of complex mental health difficulties increased 2.029% to 2.032%.
- The accuracy (a.k.a. hit rate) of a rule classifying none of study-population records as meeting our definition of complex mental health difficulties decreased from 97.971 to 97.968%.
- The odds that record did not meet our definition of complex mental health difficulties remained unchanged at 48-times as likely.

12. Line 190: Could the authors justify the age 30 as a cut off for “antecedent” events? If a patient has an “antecedent” event such as ADHD under 30, do they automatically also have a concurrent event for ADHD? As obviously diagnoses such as these may be recorded by the GP very infrequently, but still be present.

We recognise the reviewer’s point about features present across the life-course. Crucially, the unit of analysis in our study is the record, not the person or healthcare phenomena. For the example given by the reviewer, the signal we are interested in is a recorded diagnosis of ADHD before the age of 30, rather than the presence of the patient’s ADHD. The patient’s ADHD will have existed undiagnosed before the diagnosis and will continue to exist afterward. Precise dates of onset (and magnitude of) mental or psychological conditions are not possible so, rather than assume diagnoses are a proxy for such things, we have tried to be clear that we are studying records rather than patients. Additionally, any age threshold is arbitrary; we used <18 as child or young person, 18-29 as emerging adult, and 30 or older as adult.

13. Line 269: Could the authors also report the exact number of controls? Some basic descriptive information on cases and controls would be useful here.

We now provide this information in a classic Table 1, as supplementary material: S4 Table. We didn’t provide a classic table 1 showing distributional summary statistics of immutable demographics of cases and controls because such information does not help us to achieve our aim. Particularly, our study adopted a predictive rather than explanatory paradigm so concerns like confounding (which some think to be highlighted in a classic table 1) are not issues (see doi: 10.1214/10-STS330). Furthermore, readers inevitably over-interpret these kinds of tables to conclude that the cases and controls from our study are representative of cases and controls, generally; that any overlap in distributions has implications for “significant” differences; and that any difference observed in our sample indicate distinguishing features. These errors are described as the Table 1 fallacy (doi: 10.2106/JBJS.21.01166) and the Table 2 fallacy (doi: 10.1093/aje/kws412) (see also "Out of balance" by Darren Dahly for a less formal discussion of the Table 1 fallacy; doi: <https://statsepi.substack.com/p/out-of-balance>).

14. Line 276: It is not clear which feature sets are shown in these figures.

Apologies for the lack of clarity. We submitted figures with accompanying metadata using the online system. It appears they were not carried through to the version sent for review. The figures were expected to be viewed with the accompanying caption. While the horizontal axis of figure 2B is labelled as average entropy, we acknowledge that the other figures are not self-explanatory. We have amended the x-axes to clearly state what the features are. For figure 2B, the updated caption is “Average entropy of did-not-attend sequences”.

15. Figure 2a: What does the true/false relate to?

As mentioned in point 14, we apologise for the inconvenience and we have amended the x-axes to clearly state that the feature is a binary indicator indicating whether the set of features defining ‘access to healthcare’ were satisfied by the patient’s record after the patient was 30 years of age.

16. Figure 3a: Is this a count of all figures?

As mentioned in point 14, we apologies for the inconvenience and we have amended the x-axes to clearly state that the feature is the count of ‘Concurrent’ family features, which are intended to represent concerning behaviour after 30 years of age, e.g. self-harm, substance misuse or dependency.

17. Figure 5: What do a, b and c refer to? I think the titles/footnotes for these figures are missing?

The reviewer is correct to note that the captions for all figures seem to have been missing from the document they received. We apologise for our part in the inconvenience. Figure 5 shows the scaled mutual information for all informative feature sets in rank order. Rank is presented in log10. A = component features. B = feature families. C = feature family combinations.

18. There is no table 4 in the manuscript, and S3 Table is not referenced anywhere.

Apologies for this error, and thanks to the reviewer for spotting it. All references to Table 5 have been replaced with reference to Table 4. Reference to Table S3 has now been included in the Methods subsection “Features and feature sets” where originally intended.

19. Table 5: While SMI is defined here, I think the use of the same abbreviation to mean two different things (severe mental illness and scaled mutual information) is going to confuse readers.

We thank the reviewer for spotting this. We have changed all uses of “SMI” as scaled mutual information to MI_scaled_.

20. Table 5: I was surprised you excluded obesity, and there was no consideration of smoking. I would have thought, alongside alcohol and drug misuse these would be useful predictors of mental health conditions. While I agree these are not specific to complex mental illness, possibly in combination with other things they may have been useful.

We agree with the reviewer and deliberated over including obesity as a feature for a considerable length of time. We summarise our two reasons for exclusion as:

1. Obesity is common and strongly socioeconomically patterned, but not specific. We have prioritised specificity over sensitivity.
2. Statistically speaking, obesity is multicollinear with other socioeconomic factors and including it stands a real risk of diluting weaker-but-specific or causally / mechanistically more-important features.

21. Line 329: While I agree there is a great deal of under-reporting/under-diagnosis in primary care; you did exclude patients with SMI and we know co-occurrence is common (around 20% in the Hardoon paper). The WHO prevalence calculated by Huang et al is in adults, whereas I think you are calculating yours over the whole population, and Huang et al estimated a prevalence far lower for Western Europe (2.4%). This study in the UK suggests around 4% which may be more accurate: <https://pubmed.ncbi.nlm.nih.gov/16648528/>. Could the authors take some of these nuances into consideration? That being said, I think the biggest under-reporting is “chronic depression”. I suspect that is because this is rarely coded for. Other colleagues have instead looked at persistence of standard depression codes over time, or long-term prescription of antidepressants. This means many with chronic depression are potentially in your control population. There are also SNOMED CT codes such as “persistent depressive disorder” which suggest chronic depression.

We thank the reviewer for the suggested reference, which we have included and commented on in the “Prevalence of complex mental health difficulties” section of our Discussion. We are grateful for the reviewer’s illustrative example of chronic depression as a likely under-recorded clinical code. We have also included this example in our list of explanations of why our observed caseness prevalence was so low.

We have taken the reviewer’s advice and included persistent depressive disorder (SNOMED-CT: 1153575004) in the set of caseness inclusion diagnoses. We apologise for our oversight in not including it as an important sibling code in the SNOMED-CT hierarchy. When we include the new code, we were surprised to note that the no records in our dataset contained a code for persistent depressive disorder (Note: we first applied the suggest change to the exclusion codelist and have presented the ramifications elsewhere in our response). We extended the search to all records within Connected Bradford and, again, found no record containing a code for persistent depressive disorder. We confirmed that persistent depressive disorder is not on the list of sensitive codes excluded by Connected Bradford, so we can only conclude that this single, childless code is not used by general practitioners in the Bradford and Airedale region.

22. Line 424: But you didn’t include “other psychosis” in your exclusions, and therefore there could well be people with psychosis in your cases cohort. The list of SNOMED codes used for incentivisation is here, if you select PCD refsets” and set Cluster ID as “MH_COD”. You will see many conditions that you did not exclude which may warrant antipsychotic prescription: <https://app.powerbi.com/view?r=eyJrIjoiNDRmYjEwMzQtZGE3MS00ZGE5LTgwMTUtNjQ2NGE1NTZiYmEzIiwidCI6IjM3YzM1NGIyLTg1YjAtNDdmNS1iMjIyLTA3YjQ4ZDc3NGVlMyJ9> .
Please, see response to comment 10.

23. General: I wonder if it warrants a comment regarding validation in other datasets?

We completely agree with the reviewer that this work requires an assessment of external validity by using another dataset. We have submitted requests to other healthcare data platforms to repeat the study elsewhere, and on a larger scale.

Reviewer #2: This manuscript studies health records to assess What features within patients’ primary-care
electronic healthcare records might indicate that a patient has complex mental health difficulties .
Complex mental health difficulties is defined as difficulties more persistent or disruptive than the common mental disorders but which do not meet current definitions of severe mental illness like psychosis or bipolar disorder. It is an interesting paper that tries to understand what distinguishes a complex case from a non-complex case.
They also used a large dataset including all patients registered for at least 1 year in a practice and have some indication of mental illness because of diagnoses or medication. Severe mental illness diagnoses were excluded.

I have problems with understanding the cases in their case-control design. Cases were those records demonstrating recent, active, complex mental health difficulties, defined by having 1) a SNOMED-CT diagnostic code for caseness from at any time prior to 31st December 2021 and 2) a prescription for medicines of interest within ten years prior to 31st December 2021.
- What happens with patients that did not have registrations for that period of time ? Because they were registered in a practice for less than 10 years? Or does the dataset also includes data from there patients from their former practices?

A patient’s historical data will follow when they move practice. It takes a week or two but the patient record at the new practice will hold the patients complete primary care data.

We selected records from patients who were registered with their general practice for at least one year because pilot work with all records indicated people with ‘cold’ diagnoses and no activity for 20+ years. We also felt that records of patients with shorter registration would likely have insufficient historical data to analyse. Thus, our decision to exclude patients based on registration was a pragmatic trade-off.

Why is this defined as cases? Why not also the ones with only a prescription \? As the authors mention GPs might not always be accurate in registering the diagnoses. So including this group with only prescription as control might not really be a good control.

The challenge in this work is differentiating between recognised cases (of the condition) and unrecognised cases (who have the condition, but it is not diagnosed / coded). For consistency of scientific reporting we use “case” to refer to the former (recognised cases) only. That means unrecognised cases are included in the controls.

We prioritised specificity of caseness rather than sensitivity to caseness. That is to say, we assume that we are less likely to suffer false positives if we apply filters for prescriptions and diagnoses, than if we applied only one of these filters. Given how weak we expected the “signal” of complex mental health difficulties to be, we reasoned that it was more appropriate to correctly identify records satisfying caseness as cases, than it was to include records that merely might be cases. Including this latter, less-certain group would likely induce greater variance in the cohort of cases and hinder our ability to identify true cases. The trade-off is that our controls will contain true cases.

Cases could also be defined as the ones with having longer period of time of either registration of diagnoses or prescriptions. As the definition suggest a persistency of symptoms

We thank the reviewer for their suggestion, which raise the question: How long is “longer”? We hope to extend our method to other datasets, in future work. We will bare the reviewer’s suggestion in mind when doing this.

The authors use an information-theoretic approach. This could explained more in the introduction; why is that innovative? They discuss it in the discussion but this could be introduced more in the first part.

We had included such an introduction in early drafts but removed it so as not to disrupt the flow of the epidemiological argument in the Introduction. We have now included a short introduction and explanation of our information-theoretic approach, of mutual information, and of its innovativeness.

They could explain more why it is important to know the features of registered complex mental health problems. As they already mentioned that registration is not accurate. So what does it tell us these features?

Our aim was to begin the journey of finding non-obvious features within patients’ records that might clarify / reduce the uncertainty about patients’ caseness status. By doing so, we hope to identify patients and respond to their needs appropriately and in a timely fashion. As the reviewer recognises, our work is based on the assumption that complex mental health difficulties are not explicitly recorded in clinical codes, and that the data are not ‘perfect’. This motivated us to find implicit codes and signals in the wider patient records. While our top-performing features cannot be said to be features of complex mental difficulties, they are features of something approximating complex mental difficulties. Our definition of caseness was deliberately conservative rather than inclusive to reduce the expected number of false positives. We also conjecture that top-performing features could constitute a working definition of complex mental health difficulties (in something like a syndromic sense) in lieu of the prevailing nosological paradigm being insufficient for the task.

The findings support the idea that complex mental health difficulties are difficult to identify and operationalise, leading to an underecording of cases that limits the use of electronic healthcare records to support identification, study, and provision of appropriate and timely care. Partly I agree that GPs under record mental health problems. But in present study about 13% had either a diagnosis or a prescription. How is that in line with prevalence in the general population?

We assume the figure of 13% to which the reviewer refers is the 13.7% of the total population within the Connected Bradford primary-care database that met our criteria for our study population. Our criteria for the study population included a record of our diagnoses of interest in addition to a record for a recent prescription of medicines of interest. This criterion is of the form “A and B” rather than “A or B”, as described by the reviewer in their comment. The distinction is important because the intersection of diagnoses and recent prescriptions will be smaller than the union of records identified by either. With this in mind, a 2023 study by Cooper et al. of the Clinical Practice Research Datalink (CPRD) Aurum primary care database suggested a 45% prevalence of the seven mental-health diagnoses that they studied (excluding bipolar disorder and schizophrenia, but including records of patients younger than 18 years of age and older than 70, for patient records across England; doi: 10.1186/s12911-023-02296-z). Our smaller prevalence might partly be explained by our requirement for records to be from patients aged between 18 and 70 years, and for records to be from patients with records of both diagnoses and recent prescriptions for particular medicines. Nevertheless, our Table 3 shows that the diagnostic criterion alone narrowed the prevalence to 2.85% (updated to 2.61% after amendments based on comments from reviewer #1). This much-lower prevalence might be explained by differences in the patient population in the Connected Bradford and CPRD databases. For example, redacted-and-rounded proportions of ethnicity data compare as follows:

| **Ethnicity** | **Cooper et al.** | **Connected Bradford** | **Percentage-point difference**  **(Cooper et al. - Connected Bradford)** |
| --- | --- | --- | --- |
|  | *(%)* | *(%)* | *(% points)* |
| White | 64.1 | 43.2 | +20.9 |
| Asian | 8.3 | 18.9 | -10.6 |
| Black | 4.1 | 2.0 | +2.1 |
| Mixed | 1.7 | 1.4 | +0.3 |
| Other | 1.4 | 2.0 | -0.6 |
| Missing | 20.5 | 32.5 | -12.0 |

Why for the concurrent feature findings or behaviours are concurrent after 30 years of age, e.g. self-harm, risk, substance misuse or dependency? What about patients younger than 30?

The reviewer correctly interprets the Concurrent-family features as relating to patients 30 years and older. We believe the reviewer is looking for the Antecedent-family features. The Antecedent family is the complement of the Concurrent family, containing all the features relating to patients younger than 30 years.
