# Supplementary material for: Identifying primary-care features associated with complex mental health difficulties
Source: PLoS One. 2025 May 8;20(5):e0322771. doi: 10.1371/journal.pone.0322771 (PMC12061163; doi:10.1371/journal.pone.0322771)
Supplement: Attachment — Submitted filename: Response_to_Reviewers_auresp_2.docx [file pone.0322771.s007.docx]

1. Line 151-154: “who have been registered with their general practice for at least one year; who, additionally, either have a record of a SNOMED-CT diagnostic code of interest within ten years prior to 31st December 2021 (see link associated with "Mental disorder | SCTID: 74732009 + child codes" in S2 Table), and who have a record of prescriptions for medicines of interest (Table 1) within ten years prior to 31st December 2021”
Should this be “either have a record of a SNOMED-CT diagnostic code… or who have a record…”, or if they needed both perhaps the word “either” needs removing?

Thank you for spotting the typo. The “either” is correct. I have changed the “and” to an “or”.

2. Line 192: “Controls’ were those records that did not meet these two criteria.”
I presume this is anyone that didn’t meet either of these criteria? So someone with recent medication but no diagnosis becomes a control as does someone with a diagnosis in the absence of medication, and people that have neither? This could be a little clearer.

Apologies for the ambiguity. ‘Controls’ were those who did not meet at least one criteria. We had a preference for specificity of cases over sensitivity. I have changed the wording to “*‘Controls’ were those records that did not meet at least one of these criteria*.”

3. Thank you to the reviewers for providing a table 1 in the supplement. While I agree these tables may be mis/over interpreted they do provide useful context; for example for other researchers who may want to do similar in other regions. I am not sure your labels match your numbers for age in this table though, with e.g. max age listed as 30/40.

I have identified the maximum age issue as a typo. I’ve corrected the order of labels in S3 Table.csv, which should have been {min, 1^st^ quartile, median, 3^rd^ quartile, max, arithmetic mean} instead of {min, max, median, Amean, quart1, quart3}. I have also corrected the labelling in the publicly-available Jupyter Notebook that creates the table (https://github.com/ConnectedBradford/CB_1759_Joining-Primary-and-Secondary-Care-/blob/main/code/UNSEEN_create_caseness_variable.ipynb).

1. The antecedent feature set includes adverse childhood experiences; however, these experiences are not defined/specified in the text. Which adverse childhood experiences were included in the antecedent feature set? The authors cite a study in the discussion which found substantial under-coding of adverse childhood events, therefore we would expect high missingness in this feature set.

Apologies for being unclear. I have replaced “*e.g. adverse childhood experiences*” with two examples of concepts that we used to represent adverse childhood experiences, “*e.g. child abuse, abandonment, etc*”. We did not define a feature for adverse childhood experiences, instead opting for multiple features indicating the variety of such experiences. Thus, I can’t check the missingness of the concept of adverse childhood experiences.

The full list of features used in the antecedent feature set are shown in the publicly-available Jupyter Notebook that details the Python code (https://github.com/ConnectedBradford/CB_1759_Joining-Primary-and-Secondary-Care-/blob/main/code/UNSEEN_create_feature_sets_appendix3.ipynb).

2. In the discussion, the authors refer to qualitative work undertaken as part of their wider work in this area and say that this was used to inform the quantitative study. From what I can see, this qualitative study is currently unpublished. The mixed-methodology should be made clearer - please add further information around how the findings from the qualitative study informed this study in the methods, for example if this informed the selection of features?

Apologies for the lack of clarity. I have now included a brief explanation within the ‘Features and feature sets’ section. In it, I explain a little more about how the interviews inspired our features, and I explain that the source of each feature is summarised in Table S3 where ‘fs_literature’ indicates a feature inspired by our literature review, ‘fs_interviews’ indicators a feature inspired by our interview study, and ‘fs_clinician’ indicators a feature inspired by the clinical members of the research team.

3. The article requires careful proof-reading for typos/phrasing issues, particularly:
o Line 55, comma needed after (dysthymia).
o Line 71, ‘,,,’ should be one comma, with a lower-case S for ‘similarly’.
o Line 137, remove ‘the’ before England.
o Line 187, removing ‘excluding those from our exclusion list’ as this phrase is redundant.
o Figure 1 title is quite long – may I suggest adding the sentences after ‘our records-based definition…’ as a footnote. Same for figs 2 and 3, after A). It seems a few of the figures have footnotes (e.g. Fig 5 includes a footnote entitled ‘10’, while others don’t). These figures are not titled/footnoted consistently, please can authors proof-read all figure titles/footnotes.
o Table 4, following ‘Active…’ should also be a footnote.
o Line 407-408, thirdly and fourthly repeated in same sentence.

I’m grateful for your keen eye. These typos have been corrected.

I respectfully disagree that “*excluding those from our exclusion list*” is a redundant phrase. Without it, the reader it not made aware that exclusions have been made.

Regarding figures and tables, I have moved the explanations to footnotes or to the main text, or removed the explanations entirely. I find it much messier and more difficult to digest the figures and tables, now; the reader has to jump around the document to piece together their understanding. The original titles might have been long but they presented all the information necessary to understand the table in one place. I request that the editor decide which style to use.

Figure 5 does not include a footnote entitled ‘10’; the ‘10’ is the base of the logarithm being referred to in the previous sentence.
